# Prediction of Parturition in Ferrets Using Ultrasonographic Fetal Measurements

**DOI:** 10.3390/ani13233707

**Published:** 2023-11-29

**Authors:** Giorgia Pettina, Fabrizia Samiani, Viola Zappone, Marco Quartuccio, Maria Carmela Pisu

**Affiliations:** 1 Department of Veterinary Sciences, University of Messina, Viale Palatucci, 13, 98168 Messina, Italy; giorgia.pettina@studenti.unime.it (G.P.); mquartuccio@unime.it (M.Q.); 2 VRC-Centro di Referenza Veterinario, 10138 Turin, Italy; fabriziasamiani@gmail.com (F.S.); mariacarmela.pisu@vierreci.it (M.C.P.)

**Keywords:** ferret gestation, parturition prediction formula, ultrasonographic parameters

## Abstract

**Simple Summary:**

Gestation in ferrets is generally 42 days (38 to 42 days). Cases of maternal and paternal cannibalism highlight the need to know the exact date of birth in order to separate the parturient mother from the other ferrets in the herd. Unlike dogs and cats, there are no established formulae for fetal ultrasound biometry and growth assessment in ferrets. The aim of this study was to develop a formula to accurately date the birth of ferret litters to prevent cannibalism and ensure proper management of the breed. The results showed a significant relationship between days to parturition and biparietal diameter, which was therefore an accurate predictive ultrasound parameter.

**Abstract:**

The gestation period in ferrets lasts 42 days (range 38–42). Numerous cases of maternal and paternal cannibalism have been reported, so it is important for breeders to know the exact date of parturition in order to separate the mother from the other ferrets in the herd. Fetal ultrasonographic biometry and specific formulae for assessing fetal growth have not yet been developed in ferrets as they have been in dogs and cats. The aim of this study was to develop a formula, similar to those available for other domestic species, that could be used in ferrets to date the birth of a litter to within one day. Among the different ultrasonographic parameters, the biparietal diameter (BP) gave a very accurate prediction and showed a significant relationship with days before parturition. The formula developed could allow better planning of care before, during and after parturition, thus helping to reduce neonatal mortality.

## 1. Introduction

Gestation in ferrets lasts 38 to 42 days and consists of a pre-implantation period of 10 to 12 days and an active gestation period of 30 days [1,2]. Pre-implantation embryos have an extended period in the oviduct at the end of which, from day 10 of gestation, they can only be retrieved from the uterus. Implantation in the ferret is centralized, with rapid invasion of the uterine epithelium by the trophoblast over a large area, which eventually becomes a zonal band of endotheliochorial placenta [2]. The corpus luteum begins to secrete progesterone immediately after ovulation, peaking between days 12 and 14 during the implantation period. Circulating progesterone levels decrease from day 24 until delivery on day 42, reaching basal concentrations about 7 days later [3]. The product of conception does not influence the duration of the luteal phase, making pregnancy and pseudopregnancy indistinguishable [4]. The gravid uterus can be visualized from day 12 of gestation and pregnancy can be diagnosed through abdominal palpation from day 14–16. Radiographic diagnosis through visualization of fetal skeletons can be performed from day 30 of pregnancy [5].

Fetal ultrasound biometry is commonly used to assess fetal growth during pregnancy in many mammalian species and involves the measurement of various structures of the uterus and fetal body [6,7,8,9]. In dogs and cats, the linear regression model is used to assess correlations between measurements of these structures and the number of days to delivery. In fact, for some anatomical structures, regression is significantly correlated with gestational age, so it is possible to determine the expected date of delivery after performing ultrasound measurements of the appropriate structures and applying mathematical formulae [10]. There are several ultrasonographic parameters that can be easily measured during pregnancy. These include, in the first half of pregnancy, internal chorionic cavity diameter (ICC), external uterine diameter (OUD) and fetal crown–rump length (CRL). In the second half of pregnancy, measurements of biparietal diameter (BP) and body diameter (BD) have been shown to be predictive in dogs and cats [9].

In the ferret, to the authors’ knowledge, there are no specific formulae available for calculating the day of parturition. The aim of this study was therefore to develop a formula, similar to those already available for dogs and cats, to accurately predict the date of parturition in ferret litters to within one day.

## 2. Materials and Methods

### 2.1. Study Design

The study was conducted from February to May 2023 and included 27 clinically healthy pregnant female ferrets (primiparous and multiparous), privately owned, aged between 24 and 60 months (40.16 ± 12.22 months), weighing between 800 and 1300 g (1028.61 ± 145.21 g), presented to the Veterinary Reference Centre of Turin for assessment of pregnancy status. Written informed consent was obtained by all owners before enrolment. Clinical history and physical examination data were recorded for each subject. All patients were in good general and reproductive health. They were divided into two groups, the first consisting of eighteen ferrets for formula design (group 1) and the second consisting of nine ferrets for formula validation (group 2).

### 2.2. Ultrasound Examination

The B-mode examination for pregnancy assessment was performed by the same operator, who had 15 years of experience in this field, using a MyLab Sigma ultrasound machine (MyLab Sigma, Esaote, Genoa, Italy) with a linear probe (7.5–15 MHz). Subjects were positioned dorsally or laterally and gently restrained without sedation, which was unnecessary to improve animal compliance. The fur over the ventral part of the abdomen was carefully wetted and moved. Alcohol and mating gel were applied to the skin. Standardized depth settings were used as far as possible and overall gain, dynamic range, focal zone and time gain compensation were optimized. A B-mode scan of the uterus was performed to assess the pregnancy status and to assess various fetal biometric parameters.

**Figure 1:**
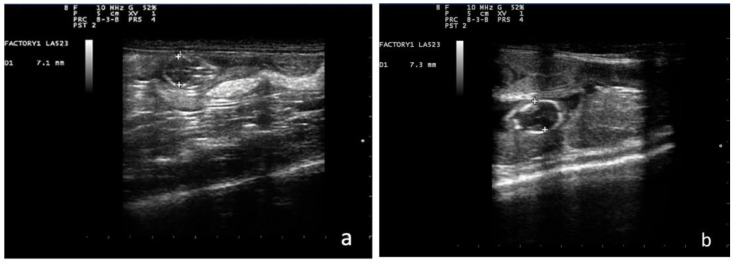
Ultrasonographic measurement of biparietal diameter (BP) in ferrets around 21 days post-fertilization: (**a**) ferrets of group 1 and (**b**) ferrets of group 2.

In the first part of the study, the fetal biometric parameter BP was obtained through a single ultrasound evaluation per animal between the 23rd and 27th day of gestation. BP was measured by drawing a line in the same transversal scan as that for crown–rump length, and the parietal bones of the skull have to be parallel in order to measure the correct distance between them [11] (Figure 1). At the same time, the visualization of fetal organs was noted. At least two BP measurements were recorded for each fetus and the mean values were calculated. The date of delivery, as reported by the owner, was then recorded.

In the second part of the study, nine pregnant ferrets underwent the same ultrasound examination, and the expected date of parturition was calculated using the formula developed in the first phase of the study.

### 2.3. Statistical Analysis

Statistical analysis was performed using GraphPad Prism 10 for Windows (GraphPad Software, San Diego, CA, USA). The relationship between BP growth and days before parturition was analyzed using a linear regression model. The growth equations were derived as y = a + bx (y = days before parturition, a = intercept coefficient, b = regression coefficient and x = measurement in mm BP), and the correlation coefficients were analyzed using Student’s *t* test (*p* < 0.001).

## 3. Results

Fetal organs were visualized during the ultrasound evaluation. In particular, the difference in echogenicity between the liver and lungs was assessed, and the kidneys, where the outline of the cortical part could be seen, and the filling and emptying of the stomach and bladder were observed.

The results of the ultrasonographic measurements of BP in the eighteen ferrets of group 1 are presented in Table 1.

**Figure 2:**
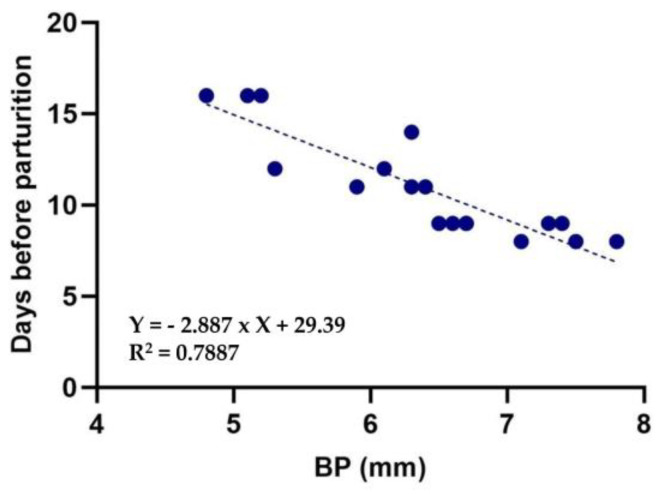
Relationship between biparietal diameter (BP) and days before parturition in ferrets.

The data obtained in the first phase of the study allowed us to develop an equation with two unknowns, finding the intercept (b) and the slope (a), *Y = (−b* × *X mm) + a*, which resulted in the following formula:

Y = (−2.887 × X) + 29.39 (R^2^ = 0.7887) (1)

where Y indicates the days before parturition (which will always be a negative number), and X indicates the mean of the biparietal diameters (mm). R^2^ represents coefficient of determination. The results of regression analysis showed a significant relationship between days before parturition and biparietal diameter (*p* < 0.001) (Figure 2). The accuracy of the formula was 94% at +/− 1 day and 100% at +/− 2 days.

In the second phase of the study, the formula was applied to nine pregnant ferrets. The expected date calculated using the formula was compared with the actual date of parturition provided by the owners, resulting in 77.77% at ±1 day and 100% with a margin of ±2 days.

## 4. Discussion

Neonatal mortality on ferret farms has been significantly reduced by modern breeding practices, but, despite this, it can still occur at alarming rates that can cause severe economic losses [12].

Accurately predicting a birth would allow close monitoring of it, which can help prevent these serious consequences of an otherwise normal birth. Over the past few decades, several studies have proposed different methods to estimate the date before parturition in dogs and cats [7,9,10,11,12,13,14,15,16,17]. More recently, ultrasound (US)-based parameters and formulae have been developed, which have shown variable accuracy, mainly related to the stage of pregnancy and litter size [11,18,19,20,21,22,23].

Ultrasound assessment of fetal and extra-fetal characteristics is essential for pregnancy staging and parturition date estimation when mating or ovulation data are not available. The most commonly studied extra-embryonic and extra-fetal parameters include inner chorionic cavity (ICC), outer uterine diameter (OUD) and placental thickness. In addition, embryonic and fetal parameters such as crown–rump length (CRL), body diameter (BD), biparietal diameter (BP), deep part of the diencephalo-telencephalic vesicle (DPTV) and kidney length are considered. The data obtained from measuring these structures have been used to develop formulae and tables for determining gestational age or days before birth (DBP) [24].

In dogs and cats, BP is the most accurate fetal parameter in late pregnancy [8] and can be detected as early as day 30 of pregnancy [6]. BP has good accuracy at 5 and 6 weeks’ gestation (95.2% and 88.4%, respectively, within ±2 days), although it is considered a reliable parameter up to 8 weeks’ gestation (85.3%, ±2 days) [22].

The aim of this study was to develop a formula, similar to those that exist for dogs and cats, that could be used in ferrets to date the parturition of a litter with only one day’s discard, thus avoiding the possibility of cannibalism, avoiding issues with males or other ferrets in the breeding group and having the best attention from the breeder for the management of the birth. The assessment of BP in the second half of the pregnancy, around the 21st day after insemination, on the other hand, provided reliable measurements. In fact, our data show that BP is a reliable indicator of gestational age, as evidenced by a coefficient of determination (R^2^) greater than 0.7. Based on the results of the measurements in the first phase of the study, it was therefore possible to develop a formula for predicting the date of delivery.

The formula predicted the date of birth with an accuracy of 94% at ±1 day and 100% at ±2 days. The accuracy at ±2 days was higher than that at ±1 day. This result was to be expected, as increasing the time from ±1 day to ±2 days increases the likelihood that the actual and predicted date will fall within the same range.

Using the formula in clinical practice, 77.77% of ferrets giving birth on the expected date within one day is not a low prediction rate, not least because in reality, the percentage at +/− 1 day should be 88.88% (8/9), as one subject gave birth a few minutes after midnight. The reason for this result is that there are a number of intrinsic and extrinsic factors which can have an influence on the time of birth.

To the authors’ knowledge, this is the first formula developed for ferret birth dating. Neonatal mortality in pets can have significant emotional and economic consequences for owners, so accurate prediction of birth date could be of great benefit to both breeders and veterinarians. The formula developed could allow better planning of care before, during and after birth, helping to reduce neonatal mortality.

## 5. Conclusions

The development of a formula for predicting ferret parturition dates represents a significant advance in the understanding of reproductive biology and may have practical applications in breeding programs and veterinary care.

In our study, assessment of BP in the second half of pregnancy provided reliable measurements. In fact, our data show that BP is an accurate indicator of gestational age, as evidenced by a coefficient of determination (R^2^) greater than 0.7.

## Figures and Tables

**Table 1:** The table shows the mean of the biparietal diameter measurements of the eighteen ferrets (group 1) used to calculate the formula.

Group 1	Biparietal Diameter (mm)	Min–Max (mm)	Day before Parturition
Subject 1	4.8	4.5–4.9	16
Subject 2	5.1	5.0–5.4	16
Subject 3	5.2	5.2–5.4	16
Subject 4	6.3	6.0–6.4	14
Subject 5	6.1	5.9–6.4	12
Subject 6	5.3	5.3–5.4	12
Subject 7	5.9	5.8–6.3	11
Subject 8	6.3	6.3–6.8	11
Subject 9	6.4	6.2–6.6	11
Subject 10	6.5	6.2–6.8	9
Subject 11	6.6	6.5–6.7	9
Subject 12	6.7	6.5–7.3	9
Subject 13	6.7	6.7–6.8	9
Subject 14	7.3	7.2–7.5	9
Subject 15	7.4	7.1–7.7	9
Subject 16	7.1	6.6–7.3	8
Subject 17	7.5	7.1–7.6	8
Subject 18	7.8	7.6–7.9	8

## Data Availability

The data presented in this study are available on justified request from the corresponding author.
